# Anacardic Acid Constituents from Cashew Nut Shell Liquid: NMR Characterization and the Effect of Unsaturation on Its Biological Activities

**DOI:** 10.3390/ph10010031

**Published:** 2017-03-16

**Authors:** Selene M. Morais, Katherine A. Silva, Halisson Araujo, Icaro G.P. Vieira, Daniela R. Alves, Raquel O.S. Fontenelle, Artur M.S. Silva

**Affiliations:** 1 Chemistry Course, Master Course in Natural Resources, University of Ceará State, Fortaleza, CE Brazil; katheasilva@hotmail.com (K.A.S.); halisson.araujo@gmail.com (H.A.); 2 Post-Graduation Course in Biotechnology, University of Ceará State, Fortaleza, CE, Brazil; icarogpv@uol.com.br; 3 Post-Graduation Course of Veterinary Sciences, University of Ceará State, Fortaleza, CE, Brazil; alves.danielaribeiro@gmail.com; 4 Curso de Ciências Biológicas, Universidade Estadual Vale do Acaraú, Sobral, CE, Brazil; raquelbios@yahoo.com.br; 5 Department of Chemistry & QOPNA, University of Aveiro, Portugal; artur.silva@ua.pt

**Keywords:** cashew nut shell liquid, anacardic acids, *Anacardium occidentale*

## Abstract

Anacardic acids are the main constituents of natural cashew nut shell liquid (CNSL), obtained via the extraction of cashew shells with hexane at room temperature. This raw material presents high technological potential due to its various biological properties. The main components of CNSL are the anacardic acids, salicylic acid derivatives presenting a side chain of fifteen carbon atoms with different degrees of unsaturation (monoene–15:1, diene–15:2, and triene–15:3). Each constituent was isolated by column chromatography using silica gel impregnated with silver nitrate. The structures of the compounds were characterized by nuclear magnetic resonance through complete and unequivocal proton and carbon assignments. The effect of the side chain unsaturation was also evaluated in relation to antioxidant, antifungal and anticholinesterase activities, and toxicity against *Artemia salina*. The triene anacardic acid provided better results in antioxidant activity assessed by the inhibition of the free radical 1,1-diphenyl-2-picrylhydrazyl (DPPH), higher cytotoxicity against *A. salina*, and acetylcholinesterase (AChE) inhibition. Thus, increasing the unsaturation of the side chain of anacardic acid increases its action against free radicals, AChE enzyme, and *A. salina* nauplii. In relation to antifungal activity, an inverse result was obtained, and the linearity of the molecule plays an important role, with monoene being the most active. In conclusion, the changes in structure of anacardic acids, which cause differences in polarity, contribute to the increase or decrease in the biological activity assessed.

## 1. Introduction

Cashews are of great economic and social importance for Northeastern Brazil. In this region, cashew farming covers an area of 670,000 ha, representing 99.5% of the Brazilian cashew culture area. The production takes place in the dry season between the harvest periods of the other species grown in the region, which gives a strategic importance in reducing fluctuations in the occupation of hand labor, mainly in the field. The cashew nut market is mainly focused on exportation [1].

**Figure 1:**
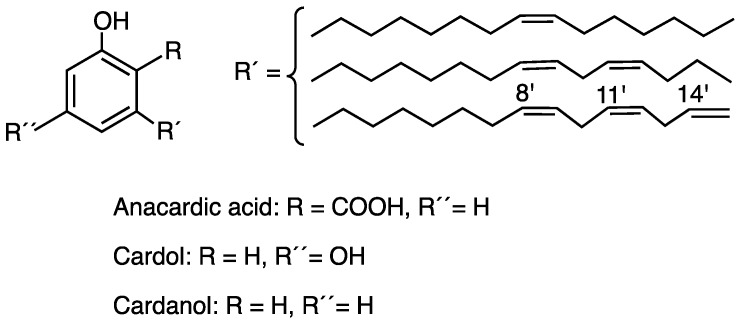
Chemical structures of the main constituents from Brazilian cashew nut shell liquid.

Cashew nut shell liquid (CNSL) is a product of little commercial value but with high technological potential due to its phenolic constitution and its various biological properties such as antimicrobial, anti-inflammatory, antitumor, antioxidant, and insecticidal properties, showing great therapeutic potential [2,3,4]. CNSL obtained by maceration with hexane at room temperature is composed by a mixture of anacardic acid, cardol, and to a lesser extent cardanol, formed by decarboxylation of anacardic acid [5]. All compounds contain a side chain of 15 carbon atoms differing in the degree of unsaturation as shown in Figure 1 [3].

In an assay of antioxidant activity of alkyl phenols in cashews, a mixture of anacardic acids (10 mg/mL) showed a higher antioxidant capacity compared to cardols and cardanols. The antioxidant capacity of anacardic acid is more related to the inhibition of superoxide generation and xanthine oxidase than the scavenging of hydroxyl radicals [6], and the C_15_-alkenyl side chain is largely associated with their activity. Due to these antioxidant functions, anacardic acid has been proposed to be a useful chemoprotectant and to have a role in skin care [7]. 

Although many studies have been conducted, the separation of anacardic acid constituents and a complete proton and carbon nuclear magnetic resonance (NMR) assignments of their three unsaturated constituents have not been completely reported. The aim of this study was to isolate the main constituents of an anacardic acid mixture obtained from natural CNSL, perform the NMR assignments, and evaluate the effect of the side chain unsaturation in the antioxidant and anticholinesterase activities and toxicity against *Artemia salina*. 

## 2. Results and Discussion

Anacardic acids, the main constituents of natural cashew nut shell liquid, are formed by a mixture of monoene, diene, and triene constituents. The long alkyl chains of anacardic acids come from the condensation of saturated or unsaturated fatty acids and phenolic compounds generated through acetate-malonate-derived pathways. Thus, palmitoleoyl-CoA can act as as start group for extention by three malonyl-CoA units, with a reduction step during chain extension and aldol cyclization that yields anacardic acid [8]. Anacardic acids are the most abundant (62.90%), and the triene component presents a higher yield, followed by diene and monoene with the same percentage (Table 1). In the high performance liquid chromatography (HPLC) analysis, a reverse phase chromatography column was used. The most polar compound is cardol triene, which was eluted first, and cardanol monoene, being the last to elute, is the least polar component.

**Figure 2:**
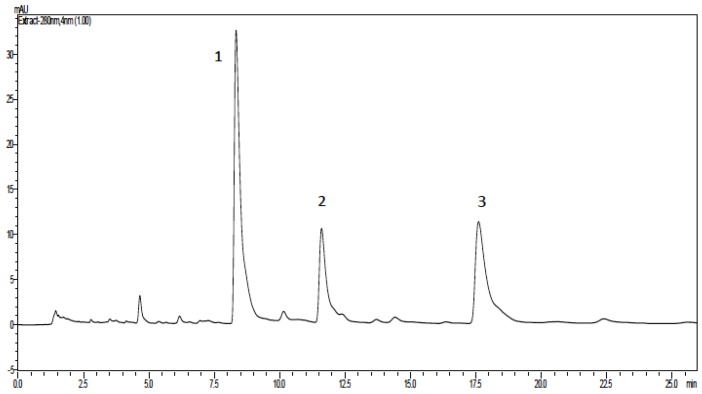
Representative high performance liquid chromatography (HPLC) profile of anacardic acids obtained from cashew nut shell liquid (CNSL).

The mixture of anacardic acids, obtained by solvent extraction from crude CNSL, showed, as expected, three main peaks in the HPLC chromatogram (Figure 2), with relative proportions of 3:1:1 for triene (1), diene (2), and monoene (3).

In another work, the CNSL was obtained and analyzed by simple normal phase HPLC—less polar-saturated anacardic acid showed lower retention times followed by monoene, diene, and triene [9]. It was found that the relative percentage of each constituent and the exact mixture depends on the species of the plant of which the 15 carbon unsaturated side chains found in the cashew plant are very lethal to Gram-positive bacteria. In comparison to our results, the percentage found for anacardic acid constituents was different from that of the India cashew plant.

Anacardic acid mixture was submitted to a chromatographic column impregnated with AgNO_3_ and each individual component was isolated and characterized by NMR spectroscopy. Complete and unequivocal proton and carbon assignments were performed and corroborated by literature data [10,11].

A chromatographic procedure for the preparative isolation of six different 6-alkylsalicylic acids (syn. ginkgolic acids), with, as alkyl substituents, C13:0, C15:0, C15:1, C17:1, C17:2, and, tentatively, C17:3 from *Ginkgo biloba* leaves, was developed [10]. The compounds were characterized by means of UV, ^1^H-NMR, and ^13^C-NMR spectroscopy. In this report, only monoene anacardic acid was characterized (C15:1). In another work [11], the monoene and diene anacardic acids were fully characterized, although spectroscopic data of triene AA was not shown.

The anacardic acids presents an aromatic moiety with absorption bands in the ^1^H-NMR spectra at ~7.36 ppm due to H-5 (t), ~6.86 ppm to H-6 (d), and ~6.75 ppm to H-4 (d); the hydrogens of the double bonds for the monoene absorb at 5.35 ppm with the integration of two hydrogens, at 5.32-5.43 ppm for the diene with integration of four hydrogens, and the triene presents three bands 5.88–5.75 (H-14′), 5.48–5.29 (H-8′, H-9′, H-11′, H-12′), and the terminal double bond at 5.05 ppm for Ha and 4.98 ppm for Hb of C15′. 

The differences in the ^13^C-NMR spectrum of the three compounds related to unsaturated carbons are as follows: the triene presents 12 sp^2^ carbons, including a terminal double bond corresponding to the absorptions in 137.05 ppm (C-14′) and in 114.91 ppm (C-15′), the last confirmed by the Distortionless Enhancement of Polarization Transfer using a 135 degree decoupler pulse (DEPT-135) as a CH_2_ group. The ^1^H-^1^H-Correlation spectroscopy (COSY) spectrum shows the correlation of the terminal CH_2_ with H-14′ and other unsaturated protons. The diene displays 10 sp^2^ carbons and the monoene shows 8 sp^2^ carbons.

The sp^3^ carbons, which absorbs in the range of 13 (CH_3_)–37 ppm, the lack of terminal CH_3_ is observed in the triene spectrum. This absence of a peak at ~0.9 ppm is also observed in ^1^H NMR.

The complete NMR data for three anacardic acids is shown below: 

*Anacardic acid monoene*: ^1^H-NMR (300 MHz, CDCl_3_) δ: 9.79 (COOH), 7.35 (1H, t, 7.7 Hz, H-5), 6.86 (1H, d, 7.7 Hz, H-6), 6.77 (1H, d, 7.7 Hz, H-4), 5.35 (2H, m, H-8′, H-9′), 2.98 (2H, t, 6.9 Hz, H-1′), 2.0 (2 × 2H, m, H-7′, H-10′), 1.61 (2H, m, H-2′), 1.28–1.32 (14H, m, H-3′, H-4′, H-5′, H-11′, H-12′, H-13′, H-14′), 0.88 (3H, t, 6.6 Hz, H-15′) ppm. ^13^C-NMR (75 MHz, CDCl_3_) δ: 176.39 (COOH), 163.57 (C-1), 111.08 (C-2), 147.91 (C-3), 122.94 (C-4), 135.44 (C-5), 116.01 (C-6), 36.59 (C-1′), 32.17 (C-2′), 29.99–29.20 (C-3′, C-4′, C-5′, C-6′, C-11′, C-12′, C-13′), 27.41 (C-7′), C-10′), 130.15–130.04 (C-8′, C-9′), 22.86 (C-14′), 14.30 (C-15′) ppm.

*Anacardic acid diene*: ^1^H-NMR (300 MHz, CDCl_3_) δ: 7.8 (COOH), 7.36 (1H, d, 7.8 Hz, H-5), 6.86 (1H, d, 7.8 Hz, H-6), 6.75 (1H, d, 7.8 Hz, H-4), 5.32–5.43 (4 × 2H, m, H-8′, H-9′, H-11′, H-12′), 2.98 (2H, t, 6.7 Hz, H-1′), 2.78 (2H, t, 5.6 Hz, H-10′), 2.04 (2 × 2H, m, 6.0 Hz, H-7′, H-13′), 1.57 (2H, q, 9.6 Hz, H-2′), 1.25–1.43 (5 × 2H, m, H-3′, H-4′, H-5′, H-6′, H-14′), 0.91 (3H, t, 9.6 Hz, H-15′) ppm. ^13^C-NMR (75 MHz, CDCl_3_) δ: 176.40 (COOH), 163.81 (C-1), 116.08 (C-2), 147.88 (C-3), 122.92 (C-4), 135.51 (C-5), 116.0 (C-6), 36.65 (C-1′), 32.21 (C-2′), 29.98-29.47 (C-3′, C-4′, C-5′, C-6′), 27.45 (C-7′), 130.36, 130.16 (C-8′, C-9′), 25.88 (C10′), 128.41, 128.26 (H-11′, H-12′), 32.21 (C-13′), 23.01 (C-14′) 13.99 (C-15′) ppm.

*Anacardic acid triene*: ^1^H-NMR (300 MHz, CDCl_3_) δ: 11.08 (COOH), 7.37 (1H, t, 7.8 Hz, H-5), 6.85 (1H, d, 7.8 Hz, H-6), 6.75 (1H, d, 7.8 Hz, H-4), 5.88–5.75 (2H, m, H-14′), 5.48–5.29 (4 H, m, H-8′, H-9′, H-11′, H-12′), 5.05 (1 Ha, dd, 10.1 Hz, c*is*, 1.2 Hz, H-15′), 4.98 (1 Hb, d, 15.9 Hz, *trans*, H-15′), 2.98 (2H, t, 7.5 Hz, H-1′), 2.81 (4H, m, H-10′, H-13′), 2.02 (2H, br t, H-7′), 1.56 (2H, m, H-2′), 1.25–1.36 (8H, m, H-3′, H-4′, H-5′, H-6′) ppm. ^13^C-NMR (75 MHz, CDCl_3_) δ: 175.58 (COOH), 163.79 (C-1), 110.77 (C-2), 147.84 (C-3), 122.91 (C-4), 135.48 (C-5), 116.05 (C-6), 36.65 (C-1′), 32.21 (C-2′), 29.97–29.45 (C-3′, C-4′, C-5′, C-6′), 27.44 (C-7′), 130.63, 129.53, 127.83, 127.07 (C-8′, C-9′, C-11′, C-12′), 31.73 (C-10′), 25.79 (C-13′), 137.06 (C-14′), 114.91 (C-15′) ppm.

The anacardic acids are interesting phenolic compounds due to their wide bioactivities, such as antibacterial [12], antioxidant, larvicide, and acetylcholinesterase activities [3]. Furthermore, it is a potent insecticide and molluscicide [13], an inhibitor of several enzymes [14,15]. Anacardic acid functions are also potent tumor angiogenesis inhibitors by targeting the Src/FAK/Rho GTPase signaling pathway, leading to significant suppression of prostate tumor growth [4]. The antioxidant properties of anacardic acid are capable of protecting human cells from oxidative stress [6,16]. This activity is due to the alkenyl chain, which is associated with the hydrophobic binding of xanthine oxidase [17]. Therefore, it is important to find the effect of the unsaturation degree in antioxidant, anti-acetylcholinesterase, toxicity, and other biological activities against *A. salina*.

The results of the antioxidant activity of three anacardic acids, assessed by 1,1-diphenyl-2-picrylhydrazyl (DPPH) radical inhibition, are shown in Table 2. The statistical analysis revealed that monoene-C_15:1_ and diene-C_15:2_ anacardic acids showed similar antioxidant action although they present lower activity relative to the triene-C_15:3_ and to standard BHT (butylated hydroxy toluene). In accordance to these results, a previous report [6] demonstrated that anacardic acid containing three double bonds in the alkyl side-chain displays greater antioxidant and enzyme inhibition capacity than the other acids possessing 1–2 double bonds. The antioxidant capacity of anacardic acid triene is more related to the suppression of superoxide generation and xanthine oxidase inhibition than to the scavenging of reactive hydroxyl radicals.

The brine shrimp lethality assay is very useful in assessing the bioactivity of plant extracts. In the investigation of Indian medicinal plants, the brine shrimp lethality assay has proven to be a convenient system for monitoring biological activities. Out of the several plant extracts screened for toxicity against brine shrimp, some species showed LC_50_ values less than 100 μg/mL, and these results support to their many medicinal activities [18]. The toxicity against *A. salina,* displayed in Table 3 is expressed by the LC_50_, which is the concentration that inhibits 50% of *A. salina* nauplii, lower LC_50_ values correspond to higher bioactivity, and the triene anacardic acid was the most bioactive. Activities of compounds are manifested as toxicity to the shrimp.

Partial inhibition of acetylcholinesterase (AChE) activity in the brain, obtained with a number of inhibitors including carbamoyl esters, has been shown to have therapeutic benefits. AChE inhibitors that penetrate the blood–brain barrier increase the levels of endogenous acetylcholine and are useful in the symptomatic treatment of Alzheimer’s disease. In a previous work, anacardic showed better action than cardanol and cardol against acetylcholinesterase and antioxidant enzymes [6], and, among the double bonds in the side chain, the triene anacardic acid has the best inhibition (1 cm), followed by diene (0.8 cm) and monoene (0.6 cm), when compared to standard physostigmine (0.9 cm). This inhibition order is demonstrated in the enzyme-linked immunosorbent assay (ELISA) measurement where triene anacardic acid has the best inhibition, followed by diene and monoene, but the statistical analysis revealed that the triene and diene are similar to standard physostigmine (Table 3).

In the antifungal activity, the results of which are shown in Table 4, the monoene anacardic acid showed higher antifungal activity since it presented the lowest inhibitory concentration in relation to the triene and diene, which are similar (*p* < 0.05). The antifungal activity is directly related to lipophylicity of the antifungal agent, an important characteristic for penetrating lipophilic microorganism cell membranes. The retention time in the reversed phase HPLC is indicative of compound polarity, since more polar compounds present lower retention times. The monoene, the more active antifungal compound, showed a higher retention time (17.59 min) followed by diene (11.59 min) and triene (8.32). The calculation of LogP also confirms the differences in lipophilicity with an increasing number of double bonds, which makes the molecule less linear and consequently more polar.

A quantitative relationship between the lipophilicity and antifungal activity of some benzoxazole derivatives against *Candida albicans* was investigated by using quantitative structure–activity relationship (QSAR) analyses. The descriptors which describe numerically the lipophilicity, logP, were calculated using Chem-Office Software version 7.0 (PerkinElmer, Boston, MA, USA) The results of this study indicate that the lipophilicity parameter has a significant effect on antifungal activity of this class of compounds [19]. This report reassures that the monoene anacardic acid, which is more lipophilic, presents higher antifungal activity.

In conclusion, the activities assessed change with the number of double bonds on the anacardic acid structure, which cause differences in their polarity. Antioxidant activity of anacardic acid components, measured via the DPPH test, increases as the polarity increases; nevertheless, in the antifungal activity, there is an inverse relationship. In Brine shrimp lethality test (BSLT) and AChE inhibition assays, the triene individual was more active, which also correlates with the increase in molecule polarity.

## 3. Experimental Section

### 3.1. General

HPLC analysis was performed on the constituents of anacardic acid using a gas chromatograph Shimadzu SPD-10VP (Shimadzu Corporation, Tokyo, Japan), with a detector UV-VIS, and the following chromatographic conditions: column Hypersil GOLD 25 cm, running time 25 min, wavelength 280 nm, flow rate 1.75 mL/min, mobile phase acetonitrile (80%) with 1% acetic acid (20%). ^1^H and ^13^C-NMR were recorded on a Bruker Avance DRX-500 (500 MHz for ^1^H and 125 MHz for ^13^C). Silica gel 60 (Merck, kiesegel 60 F254, 0.20 mm) were used for analytical TLC. Silica gel 60 (Merck, 230–240 mesh) was used for column chromatography. All compounds were visualized on TLC by spraying with vanillin/perchloric acid/EtOH followed by heating.

### 3.2. Cashew Nut Shell Liquid 

The cashew nut shells were separated from nut and then macerated twice with hexane at room temperature for 24 h. The hexane extracts were combined and evaporated to dryness, leaving behind the cashew nut shell liquid, a viscous dark liquid with a 4.5% yield in relation to whole cashew nut [5].

### 3.3. Extraction of Anacardic Acid from Cashew Nut Shell Liquid

Natural CNSL (100 g) was dissolved in methanol and 5% water, and 50 g of calcium hydroxide were slowly added. The formed calcium anacardate precipitate was filtered, washed with methanol, and then heated to 45–50 °C for 3 h, and it was then suspended in distilled water and 11 M HCl, extracted with ethyl acetate, and dried with dry sodium sulfate. The solvent was evaporated to yield anacardic acid with 60% yield. The constituents were identified by comparison with standards by HPLC.

### 3.4. Separation of CNSL Constituents In Silica Gel Chromatographic Column Impregnated with Silver Nitrate (AgNO_3_)

The mixture of anacardic acids (30 g) was submitted to column chromatography using 300 g of silica gel impregnated with AgNO_3_. The silica gel was mixed with AgNO_3_ (32.5 g), dissolved in 125 mL of water in a vessel that was covered with aluminum foil, and then dried in an oven at a temperature of 75 °C for three days prior to use [20]. The anacardic acid (30 g) was mixed with 30 g of silica, forming a powder mixture. This mixture was added to the top of a chromatographic column containing 300 g of silica impregnated with silver nitrate. The column was eluted initially with hexane, and the eluent was then a mixture of hexane, ethyl acetate and methanol in proportions of increasing polarity. Thus, 214 fractions were obtained, and, after analysis by thin layer chromatography (TLC), the following fractions were pooled: 51–62; 63–66; 67–87; 142–159; 160–169; 170–214. These were analyzed by HPLC and subsequently selected for new columns to obtain the three anacardic acids.

### 3.5. Antioxidant Activity Was Determined by a Spectrophotometric Procedure

The samples (0.1 mL of methanol solutions) at concentrations ranging from 10,000 to 1 ppm, were mixed with 3.9 mL of 6.5 × 10^−5^ M DPPH in methanol, and the UV absorbance of the reaction mixture was read at 515 nm after 1.0 h. To calculate the DPPH inhibition percentage (IP%), the following equation was used:

$$\mathrm{IP}=\frac{A_{\mathrm{DPPH}}-A_{\mathrm{Sample}}}{A_{\mathrm{DPPH}}}\times 100$$

where *A*_DPPH_ is the absorbance of the DPPH solution, and *A*_Sample_ is the absorbance of the solution containing the extract at a particular concentration. The inhibitory potential (%) of each sample concentration was applied in the Microsoft excel program to calculate the 50% inhibitory concentration (IC_50_), by linear regression analysis (concentration X IP%). The results were compared with that of quercetin, the standard antioxidant [3].

### 3.6. Quantitative Evaluation of AChE Inhibition by Microplate Assay

AChE activity was measured using a modified 96-well microplate assay based on Ellman’s method [21] and modified by Rhee [22]. Such an extremely sensitive method is based on measuring thiocholine production where acetylthiocholine is hydrolyzed. This is accomplished by the continuous reaction of thiol with 5,5′-dithiobis (2-nitrobenzoic acid). Solutions: A. Tris/HCl 50 mM, pH 8; B. Tris/HCl 50 mM, pH 8, with 0.1% bovine albumin fraction V; C. Tris/HCl 50 mM, pH 8, with NaCl (0.1 M) and MgCl_2_·6H_2_O (0.02 M).

To each well of a 96-well microplate, 25 μL of acetylthiocholine iodide (15 μM), 125 μL of 5,5′-dithiobis (2-nitrobenzoic acid) in Solution C (3 μM DTNB or Ellman's reagent), 50 μL of Solution B, and 25 μL of compound dissolved in MeOH and diluted in Solution A at concentrations of 1.56, 12.5, 25, 50, 100, 200, and 400 μg/mL were added. The absorbance was measured at 405 nm for 30 s. Afterwards, 25 μl of the enzyme AChE (0.22 U/mL) was added, the absorbance was again read every 5 min of incubation four times. The percentage of AChE inhibition was calculated by comparing the reaction rates of samples to the negative control (10% MeOH in Solution A, considering 100% as the total activity of AChE). Physostigmine was used as a standard at concentrations ranging from 1.56 to 400 μg/mL. A BioTek ELISA reader (model ELX 800, software “Gen5 V2.04.11”, BioTek Instruments, Inc., headquartered in Winooski, VT, USA) was used to determine reaction rates. This analysis was carried out in triplicate (n = 3) to calculate the mean and standard deviation.

All samples were analyzed with 3 replicates for each triplicate, obtaining 9 sets of absorbances per sample that were analyzed as follows—after normalization of the data, a non-linear regression curve test was performed by the GraphPad Prism statistical program (GraphPad Software, Inc., La Jolla, USA).

### 3.7. Assessment of Anticholinesterase Activity by TLC

This bioassay consists in the application of the samples to TLC plates and spraying the plate with Ellman’s reagent, which was prepared by mixing 5,5′-dithiobis-2-nitrobenzoic acid (DTNB) and a buffer solution of acetylthiocholine iodide (ATCI). The TLC plate was subsequently sprayed with AChE enzyme (3 U/mL). After 3 min, enzyme inhibition is observed by the presence of white spots on the yellow plate. The TLC enzyme test is basically qualitative but is still significantly sensitive. The following solutions were prepared for this test: (1) 50 mM Tris/HCl pH 8 (buffer); (2) 50 mM Tris/HCl pH 8 containing 0.1% bovine serum albumin (BSA); (3) 1 mM Ellman’s reagent; and (4) 1 mM ACTI. The lyophilized enzyme AChE was diluted in Solution (1) to prepare a 1000 U/mL enzyme solution. Five-microliter aliquots of compounds in CHCL_3_ (4 mg/mL) were initially applied to TLC plates (DC-Alufolien, silica gel 60 F254, 0.2 mm Merck). The plate was then sprayed with Solutions (3) and (4). After 3 min, which is the time necessary for the solution to completely dry, the plate was sprayed with AChE (3 U/mL). After approximately 10 min, the appearance of white spots was observed and their diameters were immediately measured. Physostigmine was used as positive control.

### 3.8. Antifungal Activity

The experimental design was a randomized block design using *T. rubrum* strains as blocks. The treatments were the three compounds (triene, diene, and monoene) at concentrations of 2.5 to 0.037 mg/mL in 6 replicates taking into account that 3 strains of the dermatophyte were used and that the experiment was performed in duplicate [23].

### 3.9. Brine Shrimp (Artemia salina) Lethality Assay

The A. salina eggs hatched in water with salinity of 12 ppm and after 48 h, the larvae were collected for bioassays. Dilutions of samples and a blank test were prepared in 0.5 mL of dimethyl sulfoxide (DMSO) in sea water. Triplicate sample solutions were prepared to be tested at concentrations of 1000, 100, 10, and 1 ppm. Ten Artemia larvae were added to each jar, and the counting of surviving larvae was made 24 h later [24].

### 3.10. Statistical Analysis

All experiments on the antioxidant effect were calculated as means ± standard deviation (SD). The one-way analysis of variance (ANOVA) test was used to determine the statistical differences followed by Newman–Keuls multiple comparison test in GraphPad Prism at 5% probability. The Microsoft Office Excel program was used for the calculation of the LC50.

## Figures and Tables

**Table 1:** High performance liquid chromatography (HPLC) analysis of natural cashew nut shell liquid.

Peak Number	Constituent	Retention Time (min)	Yield (%)
1	Cardol triene	4.41	15.36
2	Cardol diene	5.75	6.96
3	Anacardic acid triene	7.48	28.00
4	Cardol monoene	8.43	1.66
5	Cardanol triene	8.83	2.96
6	Anacardic acid diene	10.35	17.77
7	Cardanol diene	12.53	2.29
8	Anacardic acid monoene	15.93	17.13
9	Cardanol monoene	20.03	1.74

**Table 2:** Antioxidant activity of anacardic acids from Brazilian cashew nut shell liquid (CNSL).

Sample	IC_50_ (mg/mL)
Monoene anacardic acid	2.06 ± 0.28 ^a^
Diene anacardic acid	1.78 ± 0.01 ^a^
Triene anacardic acid	0.81 ± 0.13 ^b^
BHT	0.266 ± 0.005 ^c^

Different letters means significant differences between samples. ANOVA and Tukey’s Multiple Comparison tests were used at *p* < 0.05. BHT = Butylated Hydroxy Toluene.

**Table 3:** Biological activities of anacardic acids from Brazilian CNSL in brine shrimp lethality and acetylcholinesterase (AChE) inhibition assays.

Constituents	BSLT * LC_50 _ (µg/mL)	AChE Inhibition Zone (cm)	AChE Inhibition (µg·mL^−1^)
Monoene anacardic acid	347.65	0.6	6.345 ± 0.532 ^d^
Diene anacardic acid	206.25	0.8	1.771 ± 0.416 ^a,c^
Triene anacardic acid	109.71	1.0	0.980 ± 0.271 ^a,b^
K_2_Cr_2_O_7_ (control)	11.01	-	-
Physostigmine	-	0.9	1.149 ± 0.046 ^a^

* BSLT = Brine shrimp lethality test. (-) test not performed. Different letters mean statistical differences at *p* < 0.05.

**Table 4:** Minimum inhibitory concentrations (MIC) and Minimum Fungicidal Concentrations (CFM) of anacardic acids against fungi *Trychophyton rubrum*.

Compound	MIC (mg/mL)	MFC (mg/mL)	LogP
Anacardic acid monoene	0.2567 ^b^	0.5167 ^b^	8.29
Anacardic acid diene	0.6733 ^a^	1.3553 ^a^	7.61
Anacardic acid triene	0.6733 ^a^	1.3500 ^a^	6.85
C.V. (%)	40.59	40.47	-

^a,b^ Means followed by different letters in the column differ statistically from one another by the SNK test, at 5% probability. C.V. (%) = Coefficient of variation.
